# Supplementary material for: Identification of photoperiod-induced specific miRNAs in the adrenal glands of Sunite sheep (Ovis aries)
Source: Front Vet Sci. 2022 Jul 22;9:888207. doi: 10.3389/fvets.2022.888207 (PMC9354845; doi:10.3389/fvets.2022.888207)
Supplement: Supplementary file 1 [file Data_Sheet_1.ZIP › Supplementary file/miRDeep2 file.zip!/miRDeep2 file/Know_Structure/SPLP42A2/oar-mir-3957.pdf]

miRBase precursor : oar-mir-3957
Total read count : 119
oar-miR-3957-5p read count : 94
oar-miR-3957-3p read count : 24
remaining reads : 1

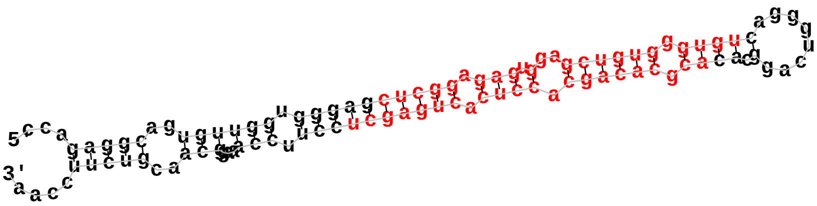

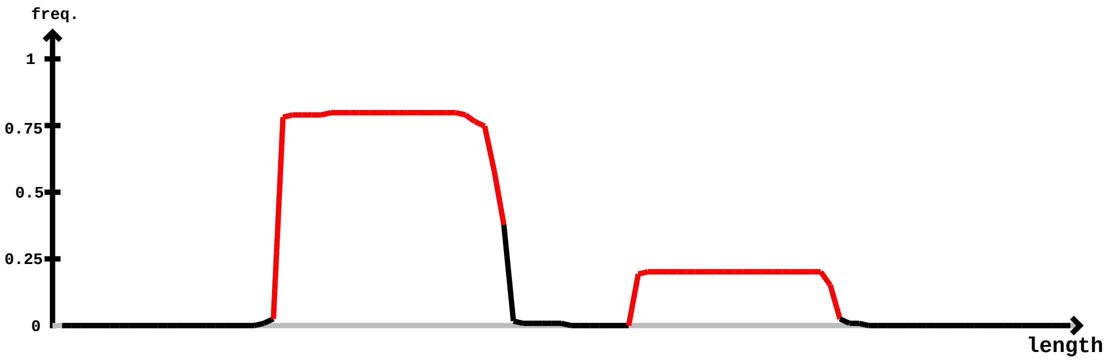


```
                                oar-miR-3957-5p
                                                                  oar-miR-3957-3p
5'- ccagaggcaguguuggugggagcucggagaguggagcugugggugucagggucaggcacacgcacagcaccucacugagcuccuuccaaaggcaacgucuuccaa   -3'    exp
    ...((((..((((((.((((((((((.(((.(..((((((.(((((........).)))).)))))).)))).))))))))))).)))...)))..)))))....   reads      mm         sample
    ....................agcucggagaguggagcugug................................................................   1          0          seq
    .....................gcucggagaguggagcugugggu.............................................................   1          0          seq
    .....................gcucggagaguggagcugugggugu...........................................................   1          0          seq
    ......................cucggagaguggagcugugg...............................................................   3          0          seq
    ......................cucggagaguggagcuguggg..............................................................   2          0          seq
    ......................cucggagaguggagcugugggu.............................................................   19         0          seq
    ......................cucggagaguggagcugugggug............................................................   24         0          seq
    ......................cucggagaguggagcugugggugu...........................................................   41         0          seq
    ......................cucggagaguggagcugugggugcc..........................................................   1          0          seq
    .......................ucggagaguggagcugugggugu...........................................................   1          0          seq
    ...........................agaguggagcugugggugucagggu.....................................................   1          0          seq
    ...........................................................acgcacagcaccucacugag..........................   6          0          seq
    ...........................................................acgcacagcaccucacugagc.........................   15         0          seq
    ...........................................................acgcacagcaccucacugagcu........................   2          0          seq
    ............................................................cgcacagcaccucacugagcucc......................   1          0          seq
```
